# Moiré pattern from a multiple Bragg–Laue interferometer

**DOI:** 10.1107/S0909049511047078

**Published:** 2011-11-25

**Authors:** Kenji Hirano, Tomoe Fukamachi, Yoshinobu Kanematsu, Sukswat Jongsukswat, Riichirou Negishi, Dongying Ju, Keiichi Hirano, Takaaki Kawamura

**Affiliations:** a Saitama Institute of Technology, 1690 Fusaiji, Fukaya, Saitama 369-0293, Japan; b Institute of Material Structure Science, KEK-PF, High Energy Accelerator Research Organization, Oho, Tsukuba, Ibaraki 305-0801, Japan; c Department of Mathematics and Physics, University of Yamanashi, Kofu, Yamanashi 400-8510, Japan

**Keywords:** moiré, interferometer, interference fringe, Bragg–Laue diffraction, dynamical theory, coherency

## Abstract

In X-ray section topography of Si 220 diffraction in a multiple Bragg–Laue mode, a moiré pattern is observed when the incident beam is divided into two parts by inserting a platinum wire in the middle of the beam. The moiré pattern can be explained by the summation of two interference fringes corresponding to the two incident beams. The coherency of the X-rays from the bending-magnet beamline is estimated using the moiré pattern.

## Abstract 2

A moiré pattern is observed by dividing the incident beam into two and formed by multiple Bragg–Laue interference fringes corresponding to the two incident beams. The coherency of X-rays from a bending-magnet beamline is evaluated using the moiré pattern.

## 1. Introduction

**Figure 1:**
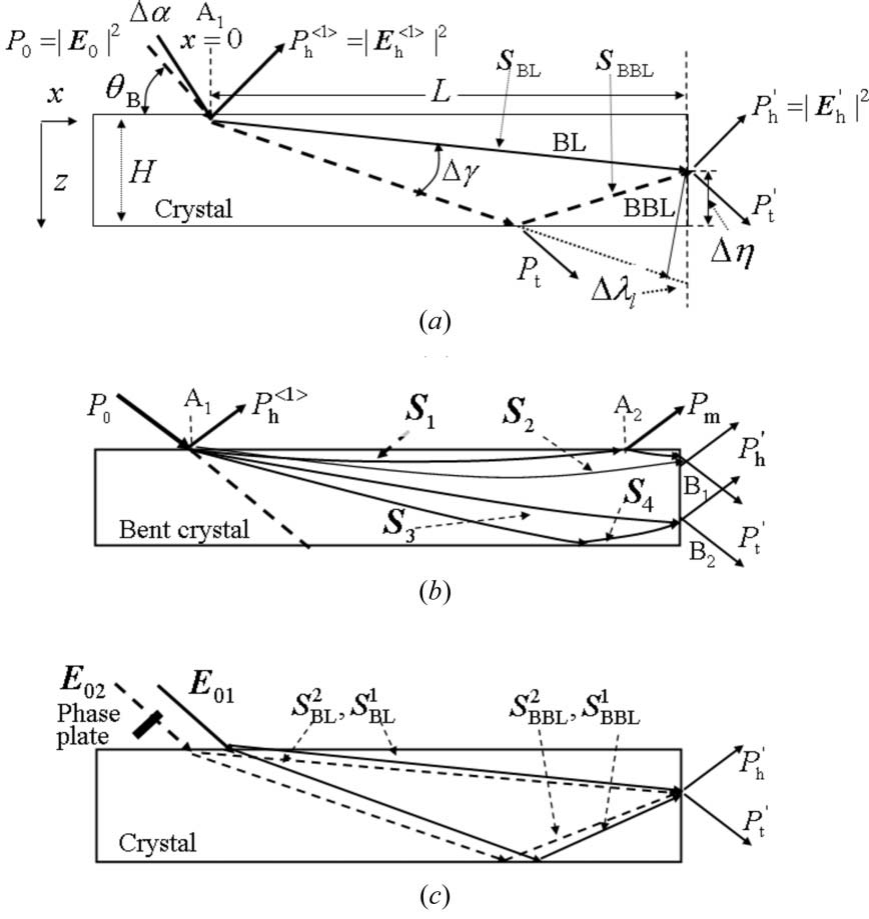
Schematic illustrations of the beam geometry in a plane-parallel single crystal (*a*) in an unbent crystal, (*b*) in a bent crystal, and (*c*) in a crystal for a two-beam MBL interferometer. 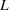 is the distance between the incident point of the X-rays and the crystal edge, and *H* is the crystal thickness. 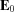 and 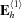 are the electric fields of the incident beam and the diffracted beam. 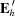 is the electric field of the diffraction beam in the direction of the diffracted beam at the exit point (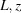) from the lateral surface. The refracted beams in the BL and BBL modes are denoted by the Poynting vectors 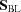 and 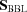, respectively. In (*c*) the second subscript 1 (2) of the electric field 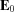 and the Poynting vectors 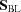 and 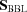 denotes the beam passing through the upper (lower) side of the platinum wire.

Interference fringes have been observed in the diffraction from the lateral surface of a plane-parallel crystal (Fukamachi *et al.*, 2004 ▶, 2005 ▶) when X-rays are incident on the surface at the incident angle where the anomalous transmission (Borrmann) effect is dominant. Under this condition the refracted beam in the crystal disperses widely from the direction of the diffraction plane to that of the transmitted beam even when the dispersion angle of the incident beam (Δα) is less than 1 arcsec. The refracted beam refers to the beam corresponding to the Poynting vector (Yoshizawa *et al.*, 2008 ▶). The diffraction geometry of the incident beam is the Bragg mode and that of the emitted beam is the Laue mode, *i.e.* the diffraction geometry as a whole is the Bragg–Laue mode. The dispersion angle (Δγ) of the refracted beam is approximately the same as the Bragg angle (θ_B_) and the ratio of the dispersion angles Δγ/Δα is of the order of 10^4^–10^5^ (Authier, 2001 ▶). The amplification of the dispersion angle is quite large and the crystal works as a lens. Since the refracted beam propagates as a spherical wave, interference between the refracted beam in the Bragg–Laue (BL) mode (its electric displacement field **D**_BL_) and that in the Bragg–Bragg–Laue (BBL) mode (its electric displacement field **D**_BBL_) occurs (Hirano *et al.*, 2008 ▶, 2009*a* ▶,*b* ▶; Fukamachi *et al.*, 2011*a* ▶), as shown in Fig. 1(*a*) ▶. If the distance from the incident point of the X-rays to the lateral surface is long, multiple diffractions in Bragg mode occur. Then the interference fringes observed in this mode are known as multiple Bragg–Laue (MBL) interference fringes. A MBL interferometer has been developed using MBL interference fringes. It works as a monochromator with high angular resolution, a beam splitter and an analyzer for a two-beam interferometer [see Fig. 1(*c*) ▶], as pointed out by Fukamachi *et al.* (2011*b* ▶).

In this paper we report that a moiré pattern has been observed by using an MBL interferometer and by inserting a platinum wire into the incident beam on the interferometer to divide the beam into two. The cause of the moiré pattern is analyzed and the coherency of the incident beam is discussed using the moiré pattern.

## 2. Experiments

**Figure 2:**
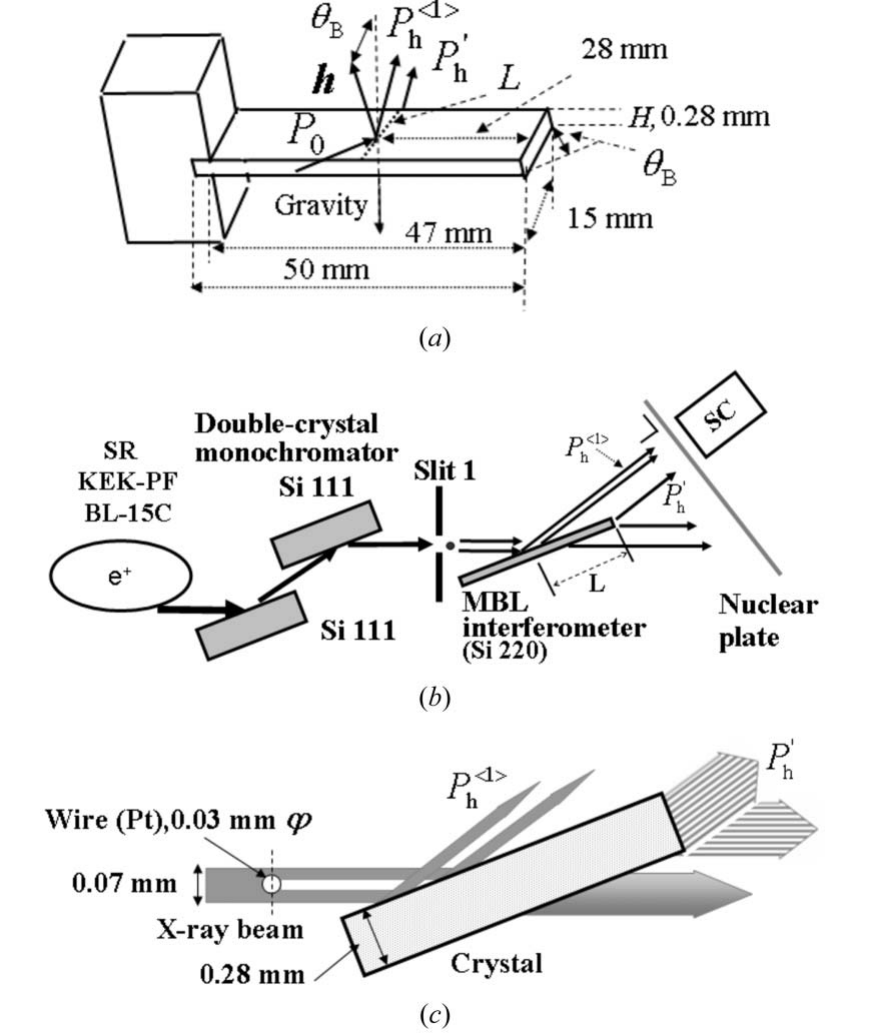
(*a*) Geometries of beams and a Si strip fixed at one end. (*b*) Schematic diagram of the measuring system. Synchrotron radiation X-rays are monochromated by a Si 111 double crystal. Diffraction intensities (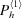 and 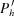) are measured either by scintillation counter (SC) or nuclear plate. (*c*) Schematic diagram of the beam geometry around the MBL interferometer.

The sample was a plane-parallel Si single crystal. The top and bottom surfaces were polished at Sharan Instruments Corporation by using the non-disturbance polishing technique. The sample size was 50 mm long, 15 mm wide and 0.28 mm thick. The beam geometry around the sample is shown in Fig. 2(*a*) ▶ and a schematic diagram of the measuring optical system is shown in Fig. 2(*b*) ▶. The Si 220 diffraction experiments were carried out using X-rays from synchrotron radiation at bending-magnet beamline BL-15C, Photon Factory, KEK, Tsukuba, Japan. The X-rays were σ-polarized and monochromated using a Si 111 double-crystal monochromator. The X-ray energy was 11100 ± 0.5 eV. The vertical width of slit 1 was adjusted to be 30 µm or 70 µm. In Fig. 2 ▶, 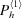 represents the intensity of the diffracted beam from the incident point, 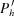 represents that from the lateral surface. The beam intensities were recorded on a nuclear plate (ILFORD L4; emulsion thickness 25 µm) and were measured using a scintillation counter (SC). Fig. 2(*c*) ▶ shows the beam geometry around the MBL interferometer.

**Figure 3:**
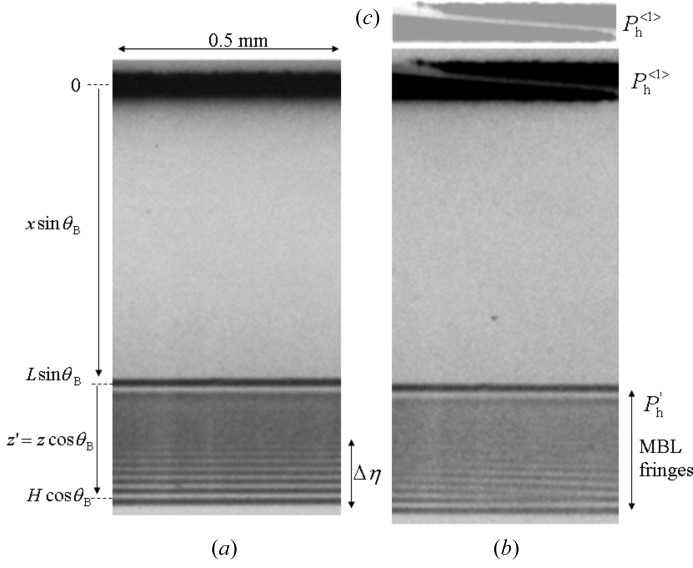
(*a*) Section topography in MBL mode taken when a single beam is incident and the vertical width of the incident beam was 30 µm. (*b*) Section topography in MBL mode taken when two beams are incident. The vertical width of the beam passing through slit 1 is 70 µm and the diameter of the inserted platinum wire is 30 µm. The distance 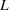 is 2.24 mm.

**Figure 4:**
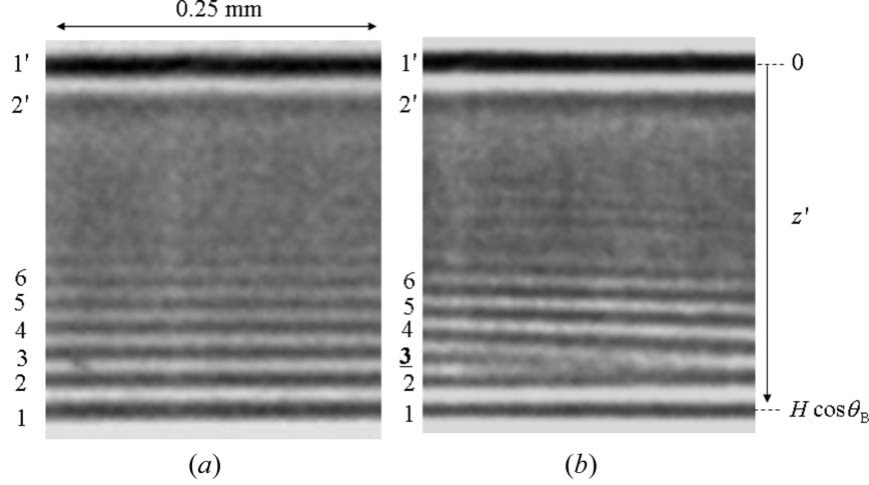
(*a*) Magnified MBL interference fringes of the lower part of Fig. 3(*a*) ▶. (*b*) Magnified MBL interference fringes of the lower part of Fig. 3(*b*) ▶.

Fig. 3 ▶ shows the section topographies of Si 220. Fig. 3(*a*) ▶ shows the topography of the primary diffracted beam 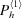 (upper) and MBL interference fringes in 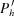 (lower) when the vertical width of slit 1 was 30 µm. In order to show the fringes more clearly, Figs. 4(*a*) and 4(*b*) ▶ show magnified photographs of the lower part of Figs. 3(*a*) and 3(*b*) ▶, in which the noise is reduced and the contrast is enhanced. Figs. 3(*b*) ▶ and 4(*b*) ▶ show topographies after inserting a platinum wire (of diameter 30 µm) in the incident beam when the vertical width of slit 1 was 70 µm as depicted in Fig. 2(*c*) ▶. In the MBL interference fringes the third fringe (dark contrast) from the bottom left disappears around the middle of the horizontal axis in Figs. 3(*b*) ▶ and 4(*b*) ▶.

## 3. Theoretical basis

The electric field 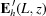 of the diffracted beam from the lateral surface shown in Fig. 1(*a*) ▶ is given by using the electric fields in the BL (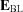) and BBL (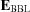) modes as
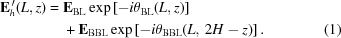
Here
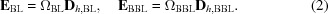

*L* is the distance between the incident point of the X-rays and the crystal edge where the beams are emitted, *H* is the thickness of the crystal, and *z* is the depth in the crystal. 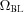 and 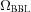 are the correction factors of the beam width on the lateral surface. 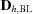 and 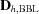 are the electric displacement fields of the diffracted beam in the BL and BBL modes, respectively. 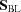 in Fig. 1(*a*) ▶ is the Poynting vector corresponding to the refracted beam and is related to the electric displacement fields as 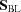 = 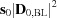 + 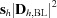. 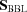 is the Poynting vector corresponding to the refracted beam and is related to the electric displacement fields by 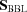 = 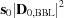 + 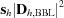. Here 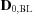 and 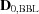 are the primary (0th) beam components of the electric displacement fields of the refracted beam in the BL and BBL modes, respectively. The phase angles in the BL and BBL modes are given by
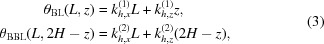
where 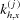 and 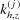 are 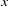 and 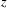 components of the wavevector of the refracted beam, respectively. The superscript (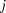) represents the branch number. The branch 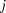 = 1 corresponds to the beam propagating in the transmitted direction such as the refracted beam in the BL mode, and 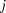 = 2 corresponds to the beam propagating in the diffracted beam direction such as that in the BBL mode. The diffraction intensity from the lateral surface is given as 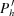 = 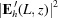.

**Figure 5:**
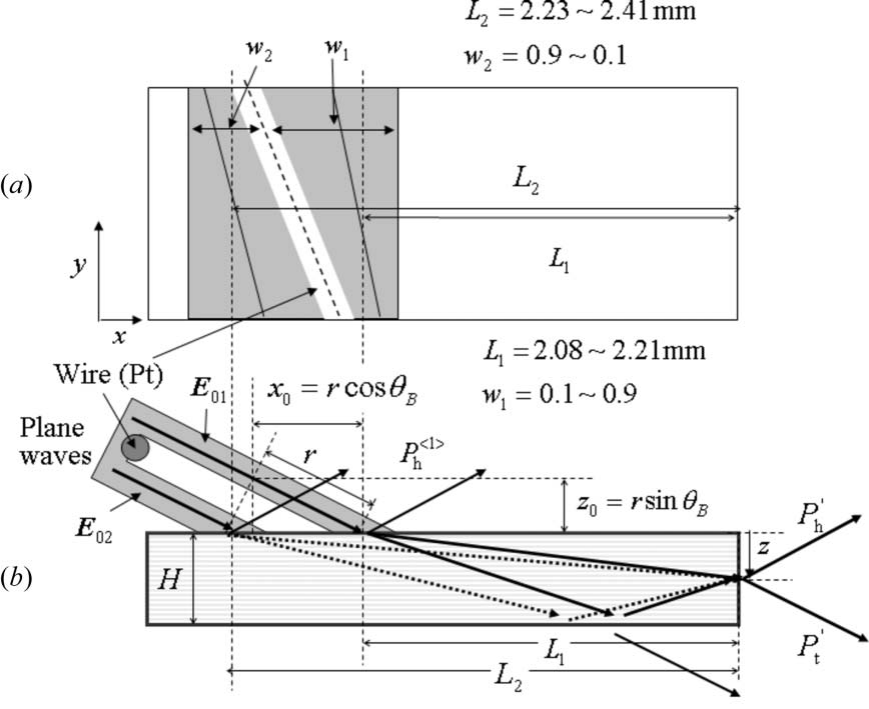
Beam geometry when the incident beam is divided into two parts by inserting a platinum wire. (*a*) Top view and (*b*) front view.

Figs. 5(*a*) and 5(*b*) ▶ show the top and side views of the beam geometry when a platinum wire is inserted into the incident beam. 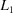 is the distance between the incident point and the crystal edge for the incident beam 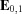 passing through the upper side of the wire, and 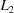 is that for the incident beam 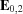 passing through the lower side. 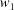 and 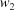 are the relative widths (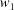 + 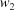 = 1) of the beams 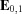 and 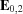. 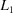, 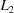, 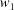 and 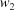 vary as a function of distance (*y*) along the crystal edge.

The electric field of the diffraction beam from the lateral surface corresponding to the incident beam 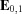 is given by
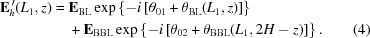
Here 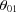 and 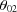 are
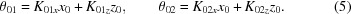


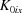
 and 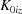 are *x* and *z* components, respectively, of the wavevector in a vacuum corresponding to the incident beam 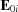 (*i* = 1, 2). 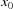 and 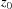 are 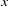 and 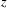 components of the path difference 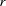 between the wavefronts of the beams 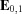 and 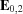, as shown in Fig. 5(*b*) ▶. The electric field of the diffraction beam from the lateral surface corresponding to the incident beam 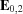 is given by
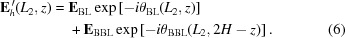
If the beams 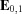 and 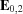 are coherent with each other, the intensity is given by
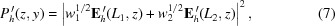
and if they are incoherent it is given by
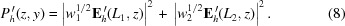



## 4. Comparison between measured and calculated results

**Figure 6:**
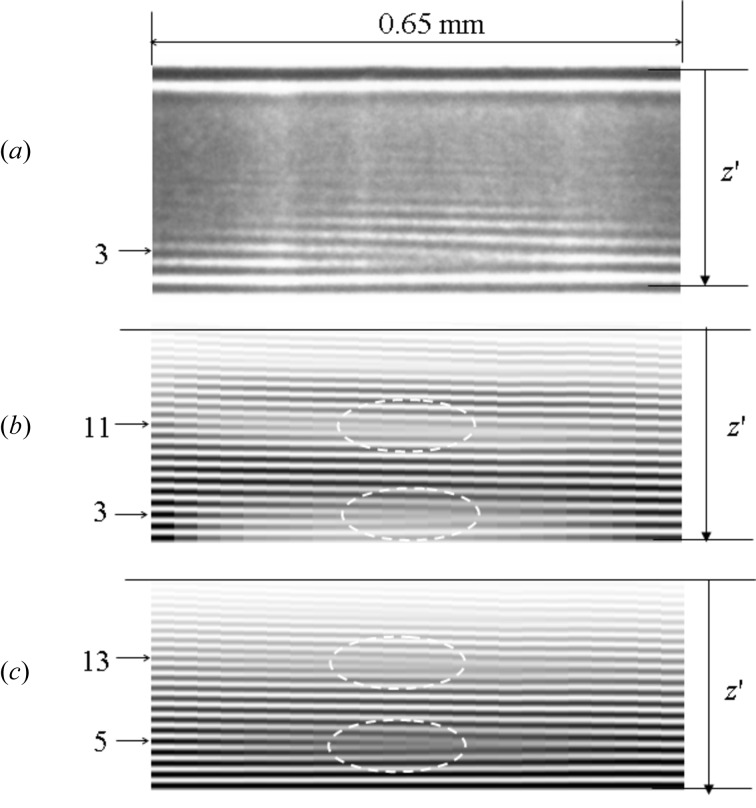
(*a*) The observed moiré pattern. (*b*) Calculated moiré pattern assuming that the two incident beams are coherent. (*c*) Calculated moiré pattern assuming that the two incident beams are incoherent.

Fig. 6(*a*) ▶ shows the measured moiré pattern, while Figs. 6(*b*) and 6(*c)* ▶ show the calculated patterns using equations (7) and (8), respectively. In the calculation, 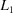, 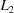, 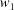 and 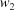 are assumed to vary linearly as a function of *y*. 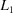 varies from 2.08 to 2.21 mm and 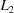 from 2.23 to 2.41 mm. 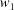 varies from 0.1 to 0.9 and 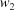 from 0.9 to 0.1. In the calculated results using equation (7) the third fringe (black contrast) from the bottom left disappears in the middle of the horizontal axis as shown in Fig. 6(*b*) ▶, while in the calculated result using equation (8) the fifth fringe disappears as shown in Fig. 6(*c*) ▶. The agreement between the measured and calculated results using equation (7) shows that the beams 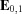 and 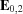 are coherent with each other. Then the optical system in Fig. 1(*a*) ▶ works as an interferometer and can be called an MBL interferometer. The disappearance of the third fringe around the middle of the figure (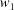 = 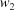 = 0.5) can be explained by the difference in the phase between 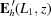 and 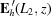 being 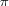 for *z* corresponding to the height of the third fringe and for 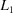 being its mean value (∼2.15 mm).

## 5. Discussion and conclusions

### 5.1. Moiré pattern

A moiré pattern is observed when the beam passing through slit 1 is divided into two incident beams on the MBL interferometer by inserting a platinum wire into the beam. The cause of the moiré pattern is attributed to be interference between two MBL interference fringes corresponding to the two incident beams 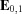 and 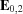, as shown in Fig. 5 ▶. In the present experiment, 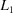 < 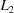 and the period of MBL interference fringes (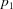) corresponding to the beam 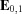 is smaller than that (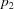) corresponding to the beam 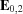. If we define the wavenumbers corresponding to the periods 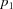 and 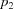 as 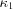 = 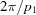 and 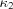 = 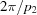, respectively, the electric fields of the diffraction beams corresponding to the incident beams 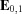 and 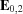 can be expressed as 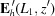 = 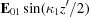 and 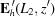 = 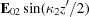 with 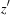 = 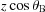. The intensity 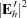 consisting of the two electric fields 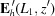 and 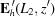 is given by
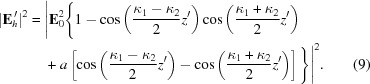
Here 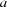 represents the degree of coherency between the two beams 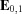 and 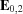 assuming that the magnitudes of the two electric fields 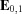 and 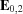 are equal to each other and are expressed by 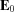. If the two beams are perfectly coherent, 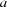 = 1, and if the two beams are incoherent, 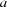 = 0. When 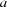 = 0, equation (9) shows an ordinary moiré pattern. It is modified by the second term in (9) when 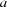 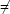 0. In Fig. 6(*c*) ▶ the fifth and 13th fringes become weak from left to right and disappear in the middle of the horizontal axis. This modulation is also a moiré pattern, but it should be a rotation moiré pattern caused by inserting the wire.

### 5.2. Coherency

We discuss the temporal (longitudinal) coherent length and spatial coherent length of the MBL interferometer. The wavevectors of the refracted beams in the BL and BBL modes are different and the paths of the two refracted beams are different, which means that the optical system works as a Michelson interferometer. It is possible to estimate the longitudinal coherent length 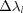 by measuring the region 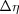 in which the interference fringes are observable as shown in Figs. 1(*a*) ▶ and 3(*a*) ▶. The coherent length 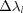 is related to 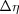 by
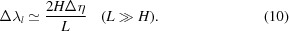
In the present experiment the resultant value of 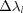 is approximately 30 µm, which is in good agreement with the value determined by the energy resolution owing to the dispersion angle of the incident beam.

The spatial coherent length can be estimated by dividing the incident beam into two parts to measure the observable range of the moiré pattern. If the observed moiré pattern is reproduced by using equation (7) or by putting 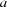 = 1 into (9), the two beams 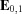 and 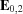 are coherent with each other. If it is reproduced by using (8) or by putting 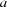 = 0 into (9), the two beams 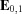 and 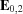 are incoherent with each other. The observed moiré pattern in the present experiment agrees with the calculated pattern using (7) and shows that the beams of 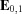 and 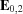 are coherent with each other. Since the distance between two beams of 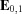 and 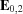 in the present experiment is 43 µm, the spatial coherent length is larger than 43 µm. According to the theoretical consideration the size of spatially coherent region 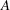 is given by 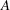 = 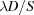, where 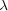 is the X-ray wavelength, 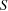 is the source dimension of synchrotron radiation, and 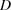 is the distance from the source point to the sample crystal. Under the present experimental conditions at beamline BL15C at KEK-PF, 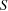 = 55 µm, 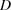 = 31 m and 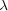 = 0.11 nm, then the size of the spatial coherent region becomes 62 µm, which is in good agreement with the estimated value using the moiré pattern.

### 5.3. Bent crystal

We can see a strong dark contrast (1′) of fringes at 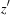 = 0 in Fig. 4 ▶. This dark contrast is not part of the MBL interference fringes, because the difference in the path lengths of the beams in the BL and BBL modes is larger than the coherence length 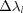 in the present experiment. This dark contrast should be caused by the interference between two mirage diffraction beams [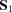 and 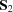 in Fig. 1(*b*) ▶] as the crystal is bent by gravity, and the paths of the refracted beams become hyperbolic forms (Gronkowski & Malgrange, 1984 ▶; Authier, 2001 ▶) and those refracted beams produce mirage fringes (Bak-Misiuk *et al.*, 1987 ▶; Chukhovskii & Petrashen’, 1988 ▶; Fukamachi *et al.*, 2010 ▶) and/or IFMRBs (interference fringes between a mirage diffraction beam and a reflected beam from the bottom surface) (Fukamachi *et al.*, 2011*c* ▶).

A moiré pattern has been observed in the MBL interference fringes when the two beams are incident on the interferometer crystal. The spatial coherent length of the beam passing through slit 1 has been estimated. It is expected that a moiré pattern owing to lattice distortion can be observed in the MBL interference fringes and should be useful for analyzing the strain around the distortion. If X-rays from an undulator beamline are used instead of X-rays from a bending-magnet beamline, the number of MBL interference fringes will be increased and detailed analysis of a moiré pattern will be possible.

## References

[bb1] Authier, A. (2001). *Dynamical Theory of X-ray Diffraction.* Oxford University Press.

[bb2] Bak-Misiuk, J., Gronkowski, J., Härtwig, J. & Wierzchowski, W. (1987). *Phys. Status Solidi A*, **99**, 345–351.

[bb3] Chukhovskii, F. N. & Petrashen’, P. V. (1988). *Acta Cryst.* A**44**, 8–14.

[bb4] Fukamachi, T., Hirano, K., Negishi, R., Kanematsu, Y., Jongsukswat, S., Hirano, Kei. & Kawamura, T. (2011*a*). *Acta Cryst.* A**67**, 154–159. 10.1107/S0108767310051573 21325718

[bb5] Fukamachi, T., Jongsukswat, S., Kanematsu, Y., Hirano, K., Negishi, R., Shimojo, M., Ju, D., Hirano, Kei. & Kawamura, T. (2011*b*). *J. Phys. Soc. Jpn*, **80**, 083001.

[bb6] Fukamachi, T., Jongsukswat, S., Kanematsu, Y., Hirano, K., Negishi, R., Shimojo, M., Ju, D., Hirano, Kei. & Kawamura, T. (2011*c*). *J. Phys. Soc. Jpn*, **80**, 083002.

[bb7] Fukamachi, T., Negishi, R., Yoshizawa, M. & Kawamura, T. (2005). *Jpn. J. Appl. Phys.* **44**, L787–L789.

[bb8] Fukamachi, T., Negishi, R., Yoshizawa, M., Sakamaki, T. & Kawamura, T. (2004). *Jpn. J. Appl. Phys.* **43**, L865–L867.

[bb9] Fukamachi, T., Tohyama, M., Hirano, K., Yoshizawa, M., Negishi, R., Ju, D., Hirano, Kei. & Kawamura, T. (2010). *Acta Cryst.* A**66**, 421–426. 10.1107/S0108767310006148 20404447

[bb10] Gronkowski, J. & Malgrange, C. (1984). *Acta Cryst.* A**40**, 507–514.

[bb11] Hirano, K., Fukamachi, T., Yoshizawa, M., Negishi, R., Hirano, Kei. & Kawamura, T. (2009*a*). *Acta Cryst.* A**65**, 253–258. 10.1107/S0108767309013968 19535846

[bb12] Hirano, K., Fukamachi, T., Yoshizawa, M., Negishi, R., Hirano, Kei. & Kawamura, T. (2009*b*). *Phys. Status Solidi A*, **206**, 1855–1859.

[bb13] Hirano, K., Fukamachi, T., Yoshizawa, M., Negishi, R., Hirano, Kei., Xu, Z. & Kawamura, T. (2008). *J. Phys. Soc. Jpn*, **77**, 103707.

[bb14] Yoshizawa, M., Fukamachi, T., Hirano, K., Oba, T., Negishi, R., Hirano, Kei. & Kawamura, T. (2008). *Acta Cryst.* A**64**, 515–518. 10.1107/S0108767308019314 18708714

